# Modulation of macrophage functions by sheeppox virus provides clues to understand interaction of the virus with host immune system

**DOI:** 10.1186/1743-422X-2-22

**Published:** 2005-03-22

**Authors:** Abdel-Aziz S Abu-EL-Saad, Ahmed S Abdel-Moneim

**Affiliations:** 1 Department of Zoology, Faculty of Science, Cairo University, Beni-Suef, Egypt; 2 Department of Virology, Faculty of Veterinary Medicine, Cairo University, Beni-Suef 62511, Egypt

## Abstract

**Background:**

Poxviruses encode a range of immunomodulatory genes to subvert or evade the challenges posed by the innate and adaptive immune responses. However, the inactivated poxviruses possessed immunostimulating capacity and were used as a prophylactic or metaphylactic application that efficiently reduced susceptibility to infectious diseases in different species. This fact is intensively studied in different genera of poxviruses. However, little is known about the basic mechanisms adopted by sheeppox virus (SPPV). SPPV causes an acute disease of sheep that recently, has been observed to reinfect its host in spite of vaccination.

**Results:**

By injecting inactivated or attenuated sheeppox virus SPPV vaccine in adult male Swiss mice, SPPV was found to reduce macrophages' functions in a local event that occurs at the site of application 12 h after vaccine administration as indicated by increased level of IL-10 and decreased level of SOD from cultured peritoneal macrophages. In contrast increased levels of IL-12, and SOD activity from cultured splenic macrophages, lymphocyte response to PHA-P, and in-vivo response to T-dependant Ag were detected. These effects were observed in both attenuated and inactivated SPPV, but more prominent in attenuated one.

**Conclusion:**

The results of this study help to elucidate, the phenomenon of existence natural SPPV infections in sheep instead of vaccination and the basic mechanisms responsible for the immunostimulating capacity of sheeppox virus. Locally, SPPV shows evidence for an immune escape mechanism that alleviates the host's immune response. Later and systemically, the virus protects the host from any fatal consequences of the immune system suppression.

## Background

Sheeppox virus, an epitheliotropic DNA virus, is classified as a member of Capripox virus genus that represent one of eight genera within the chordopox virus subfamily of the Poxviridae. Genus Capripoxvirus is comprised of sheeppox virus, goatpox virus, and lumpy skin disease virus that cause disease in sheep, goats, or cattle, respectively. These viruses are responsible for some of the most economically significant diseases of domestic ruminants in Africa and Asia [9,10]. Live attenuated SPPV and subunit formulations have been used experimentally and in enzootic as well as outbreak areas as vaccines against sheeppox, goatpox, and lumpy skin disease [8,9].

The Poxviridae are the largest known viruses [10] that have strong immunogenic properties. Poxviruses modulate the immune response in infected hosts by inhibiting the synthesis and release of IL-1 from infected cells; encoding soluble cytokine receptors for tumor TNF-α, TNF-β, IL-1, and importantly, IFN-γ; synthesizing virus-encoded cytokines like epidermal growth factor and transforming growth factor, which antagonize the effects of host cytokines mediating the antiviral process [16,26]. In addition, inducing apoptosis in a significant number of antigen-presenting cells [20] as well as inducing IL-10 release that has the capacity to impair the initiation of an acquired immune response [16,21]. If the viruses fail to secrete such immunomodulating proteins, as when the respective genes are deleted or the viruses are inactivated, the strong immunogenicity of the viruses may induce host immune reactions which are no longer inhibited [19]. This is supported by earlier studies revealing enhanced phagocytosis, natural killer (NK) cell activity, and release of IFN-α by the use of inactivated poxviruses [7,24]. Moreover, the secretion of TNF-α, IL-2, and granulocyte-macrophage colony-stimulating factor could also be enhanced [23,30]. This assumption leads to the recommendation of use inactivated poxviruses as prophylactic or metaphylactic tool in reducing susceptibility to infectious diseases [31]. However, it has been reported recently that inactivated parapoxvirus ovis, was able to induce apoptosis of antigen-presenting cells (APC) [20].

In this study, sheeppox virus-induced immunomodulating effects were characterized to elucidate the basic mechanisms responsible for understanding the interaction of SPPV with host immune system. As markers for early immunological reactions, peritoneal cells were tested after in vivo treatment with SPPV for IL-10 release and SOD activities. Markers for late reactions were the proliferation response of splenocytes to PHA-P, IL-12 release, and SOD activity, of cultured splenic macrophages from treated mice. The antibody response to CRBC was also assessed in different treated groups.

## Results

### Secretion of IL-10 by peritoneal macrophages

**Figure 1:**
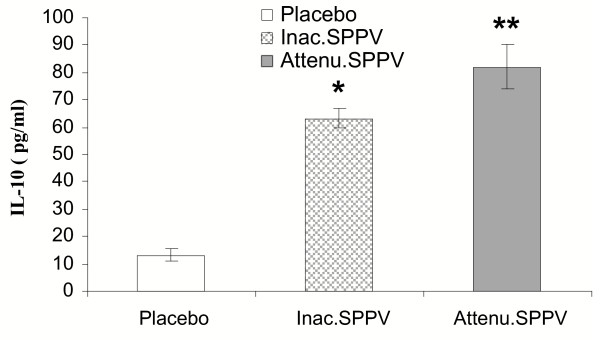
**IL-10 release from cultured peritoneal macrophages 12 h post SPPV immunization**. Mice were injected intraperitoneally with PBS, inactivated SPPV, or attenuated SPPV. Peritoneal macrophages were harvested 12 h post inoculation (five/group). Macrophages were co-cultured with LPS 1 μg/ml for 48 h, IL-10 was measured in the culture supernatant. Bars represent mean ± S:E:M: of cytokine. SPPV vaccinated mice are significantly different from controls at *P < 0.05 or **P < 0.01.

At 12 h post treatment, both vaccinated groups showed increased IL-10 (P < 0.05) in comparison to placebo. Attenuated SPPV vaccinated group showed significant (P < 0.01) increase in comparison to placebo. No significant variation was observed between the SPPV treated groups Fig. 1.

### Secretion of SOD by peritoneal macrophages

**Figure 2:**
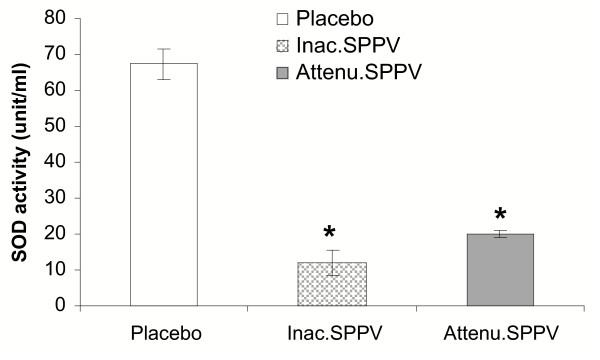
**SOD activity of cultured peritoneal macrophages 12 h post SPPV immunization**. Mice were injected intraperitoneally with PBS, inactivated SPPV, or attenuated SPPV. Peritoneal macrophages were harvested 12 h post inoculation (five/group). Macrophages were co-cultured with LPS1 μg/ml for 48 h, SOD was measured in the culture supernatant. Bars represent mean ± S:E:M: of SOD. SPPV vaccinated mice are significantly different from controls at *P < 0.05.

At 12 h post treatment, both SPPV treated groups showed significant decreased SOD activity (P < 0.05) in comparison to untreated group. No significant variation was observed between the SPPV treated groups Fig. 2.

### Secretion of IL-12 by splenic macrophages

**Figure 3:**
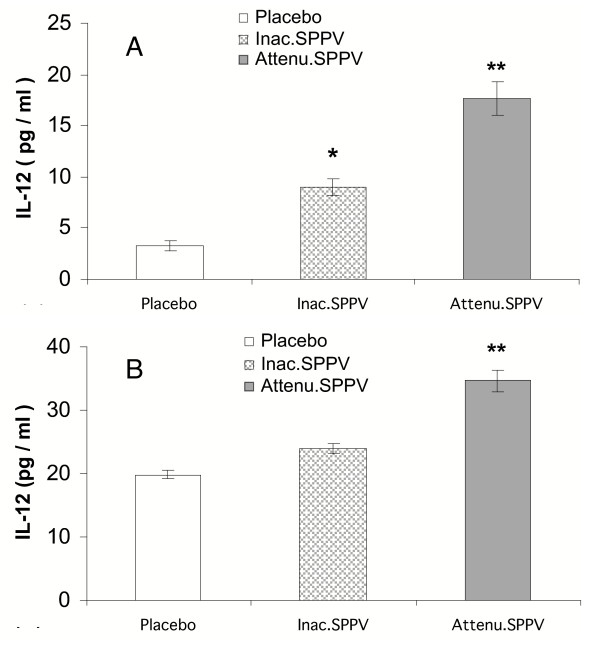
**IL-12 secretion from cultured splenocytes collected at 6 d (A) ; 9 d (B) post SPPV immunization**. Splenic macrophages were harvested from mice (five/group), cultured with LPS 1 μg/ml for 48 h. IL-12 was measured in the culture supernatant. Bars represent mean ± S:E:M: of cytokine. SPPV vaccinated mice are significantly different from controls at *P < 0.05 or **P < 0.01.

At 6 day post treatment, both SPPV treated groups showed significant increased IL-12 (P < 0.05) in comparison to untreated group Fig. 3a. Attenuated SPPV treated group showed highly significant value (P <0.01) than that recorded with placebo mice. At 9 day post inoculation attenuated SPPV treated group showed significant increased IL-12 secretion (P < 0.01) in comparison to both inactivated SPPV treated group and untreated one Fig. 3b.

### Splenocytes blastogenic response

No significant variations were detected among different groups at 12 h, 3 and 6 days post treatment. Significant increased splenocytes' proliferation response to PHA-P (P < 0.01) was observed at 9, and 12 days post inoculation in attenuated SPPV from placebo. Attenuated group showed significant increased from inactivated group at both 9 (P > 0.05) and 12 days (P > 0.01). No significant variation was observed between inactivated SPPV and control group at 12 day post treatment Table. 1.

**Table 1:** T-cell proliferation based on the MTT dye uptake method of cultured splenocytes at different intervals post SPPV immunization

Time post treatment	Treatment		
	
	Placebo	Inac.SPPV	Atenu. SPPV
12 h	1.22 ± 0.08	1.37 ± 0.14	1.53 ± 0.08
3 d	1.24 ± 0.14	1.39 ± 0.19	1.36 ± 0.18
6 d	1.36 ± 0.09	1.51 ± 0.17	1.45 ± 0.2
9 d	1.16 ± 0.05	1.53 ± 0.08*	2.18 ± 0.12**
12 d	1.78 ± 0.18	1.93 ± 0.05	2.58 ± 0.2**

Splenocytes were harvested from mice (five/group), cultured with PHA-P (10 μg/well) for 48 h, SI of SPPV treated mice are significantly different from controls at *P < 0.05 or **P < 0.01.

### Secretion of SOD by splenic macrophages

No significant variations were detected among different groups at 12 h post treatment. At 3 day post inoculation significant increased SOD activity was observed (P < 0.01) in attenuated SPPV in comparison to the other groups. Significant increased SOD activity (P < 0.05) was observed at 6 (P < 0.05), 9 (P < 0.01), and 12 (P < 0.01) days post inoculation in both SPPV treated groups in comparison to untreated group Table. 2.

**Table 2:** SOD activity of cultured splenocytes' macrophages at different intervals post SPPV immunization

Time post treatment	Treatment		
	
	Placebo	Inac.SPPV	Atenu. SPPV
12 h	13.3 ± 1.65	13.6 ± 1.22	12.8 ± 1.35
3 d	12.5 ± 2.8	10.82 ± 2.21	133 ± 23.1**
6 d	10.2 ± 1.79	107.7 ± 25 *	143.7 ± 38*
9 d	52.3 ± 19.1	253.3 ± 33.4**	240.7 ± 42.4 **
12 d	47.6 ± 8.5	266 ± 26.6**	293 ± 37.1**

Splenic macrophages were harvested from mice (five/group). Macrophages were cultured with LPS (1 μg/ml) for 48 h and SOD was measured in the culture supernatant. SPPV treated mice are significantly different from controls at *P < 0.05 or **P < 0.01.

### Immune response to CRBC

**Figure 4:**
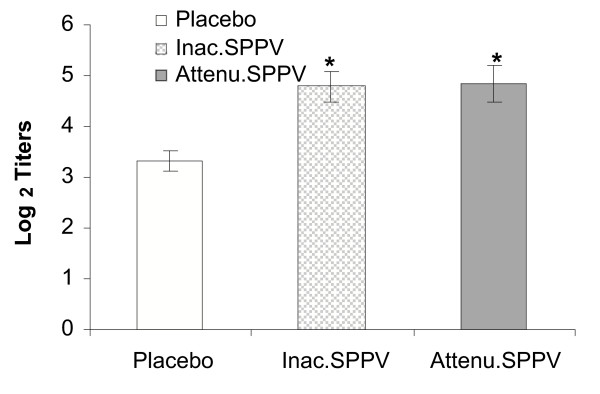
**Humoral antibody response to CRBC**. Mice were treated with live or inactivated SPPV or placebo. At 7 day, P.I., CRBC were administered by i.p route of inoculation. Seven days later, haemagglutinating abs were measured by HA test. SPPV treated mice are significantly different from controls at *P < 0.05.

Both SPPV treated groups showed significant increase in haemagglutinating antibody titers to CRBC (P < 0.05) in comparison to untreated group Fig. 4.

## Discussion

Inactivated poxviruses showed immunostimulating capacity. Such capacity is common to poxviruses of different genera [11]. This fact renders poxviruses common vectors in vaccine development. On the other hand, poxviruses express a wide variety of proteins that are nonessential for virus replication in vitro but help the virus to evade the host response to infection that may in turn impair the immunological response against live viruses. Such nonstructural proteins includes soluble receptors for IFNα, β, TNFα, SOD-like protein,..etc. [16,26,35]. These facts together with the recurrence of SPPV infection among vaccinated flocks [33] let us to study first, the possible pivotal role of SPPV in inducing local immunosuppression as a crucial mechanism of immune escape, and second, to evaluate the potential beneficial systemic effect of SPPV on the host immune system.

Early immune response to SPPV at the site of inoculation provides an explanation for the ability of SPPV to induce local suppression at site of inoculation as indicated by increased IL-10 secretion and decreased SOD enzyme activity 12 h after injection. IL-10, a prototypic anti-inflammatory cytokine, that inhibits APC function and ultimately the induction of anti-virus immunity [12], prevents the differentiation of DC from monocytes [5], and inhibits the down regulation of receptor-mediated endocytosis and macropinocytosis following exposure to a soluble immunogen [25]. In addition, IL-10 reduces the production of IL-2, IFN and TNF by murine Th1 cells [14] as well as the IL-12 production by APC [18]. IL-10 may also, intervene at the level of antigen processing within the cell so that antigen is not degraded effectively and MHC class II molecules fail to load with peptide [13]. Accordingly, enhanced secretion of IL-10 by SPPV has the capability to inhibit antigen presenting cell (APC) function as well as innate response and hence impairing the initiation of an acquired immune response. Such effect inhibits the generation of immunological memory necessary to immunity in subsequent exposure [2]. Interestingly, both inactivated and attenuated SPPV showed significant increase in the IL-10 production from peritoneal macrophages. On the other hand, decreased in vitro SOD activity of cultured peritoneal macrophages noticed in SPPV treated groups may also enhance in vivo virus survival in, and in the presence of phagocytes. Superoxide is generated deliberately by phagocytes during the respiratory burst to kill microorganisms [3]. The regulation of cellular SOD in poxvirus-infected cells might disrupt the balance of oxidants and antioxidants. Superoxide radicals arise during numerous oxidations in both living and nonliving systems and can act directly as oxidants or generate other reactive products that are toxic to cells, causing damage to lipid membranes, nucleic acid, carbohydrates, and proteins. SOD scavenges active oxygen species generated during aerobic metabolism. Consequently, aerobic existence is accompanied by a persistent state of oxidative siege, and the survival of a given cell is determined by its balance of reactive oxygen intermediates and antioxidants. Disturbance of this balance can lead to disease [17]. Further, since oxidative stress can induce apoptosis [6], this may aid virus dissemination, a fact recorded with other poxviruses [20]. Additionally, an increase in the cellular oxidant status results in activation of transcriptional factors, such as NF-κB [28], that may be necessary for replication of some viruses [27,37]. Virulent viruses of SPPV may advocate similar immune evasion mechanisms that deflect down regulation and abortion of cell-mediated immunity.

In order to gain more insight into the processes underlying the possible immune stimulating effect of vaccination with SPPV, the ex-vivo levels of IL-12, SOD in splenic macrophages, and the magnitude of splenocyte proliferative response to PHA-P as well as the in-vivo effect of SPPV on humoral immune response to TD Ag; CRBC were studied. The obtained data showed that mice successfully vaccinated with SPPV displayed significant increases in IL-12 levels at 6 and 9 days post vaccination as compared to unvaccinated mice. IL-12 was examined at that time as it is likely that a minimum of five days is required to create an effective barrier by poxvirus-mediated T-cell activation and cytokine secretion [11]. Interestingly, SOD, and lymphocyte blastogenesis as well as humoral immune response to CRBC were significantly enhanced in SPPV vaccinated mice especially in group treated with attenuated SPPV that may be related to virus replication. Enhanced splenic T-lymphocyte response to PHA-P in vaccinated mice indicates that SPPV may help in maintaining optimum T-cell responsiveness after vaccination. These effects might also rely on increased amounts of SPPV induced enhancement of IL-12 due to ability of IL-12 to induce early phase of NK and T cell activation [34]. Using CRBC as model of TD Ag, it has been shown that SPPV was capable of enhancing TD Ab responses. Interestingly, enhancement was observed in mice vaccinated with either inactivated or attenuated SPPV. Furthermore, enhancement of lymphocyte blastogenesis and SOD were also recorded. These results are the first to demonstrate an immunostimulant effect of SPPV. Enhanced responses to TD Ags in SPPV vaccinated mice may be due to the enhancement of IL-12 production, increased responsiveness of T-lymphocyte and the SOD activity that were recorded in this study. IL-12 enhancement may initiate such cascade as it has been found to be the dominant factor in development of the Th1 phenotype and also directly, or from its associated release of type-1 cytokines, enhances the activation and production of Th1-associated immunoglobulins [1]. In addition, IL-12 is not only a connective element between accessory cells and lymphocytes, but it is also a key molecule for programming the macrophage and dendritic cell functions [4]. One of the major effects of IL-12 on macrophages and dendritic cells is the induction of IFN-γ, resulting in a positive feedback capable of activating them in different situations [15].

Our observations indicate that the SPPV-induced reduced macrophages' functions in a local event that occurs at the site of application 12 h after administration. In contrast, 3 till 12 days after injection of either inactivated or attenuated SPPV, enhanced functions of splenic macrophages and increased responsiveness of lymphocytes were found to be significantly increased that was more pronounced in attenuated vaccine rather than inactivated one. The combination of suppressive and stimulatory mechanisms is a complicated blend of viral survival strategy.

## Conclusion

Locally, SPPV shows evidence for an immune escape mechanism that alleviates the host's immune response to viral proteins and therefore generates the possibility of replicating in the host in spite of vaccination. Such suggested enhanced replication strategy appears to be essential for the continued existence of SPPV. Surprisingly, injection of inactivated SPPV produced similar effect but to a lower extent that denotes: evading host immune response by SPPV is not dependent on virus infectivity. Later and systemically, the virus protects the host from any fatal consequences of the suppression of the immune system by compensatory enhanced activities of splenic macrophages and lymphocytes. This cascade of immunological events would be an excellent strategy for the virus to survive.

## Methods

### Animals

Eight-week-old Swiss male mice (Biological Supply Center, Theodar Bilharz Research Institute (TBRI), Cairo, Egypt) were used within this study. Mice were bred conventionally, and received standard laboratory diet as well as water ad libitum.

### Virus

SPPV attenuated vaccine (Vaccine and Sera Production and Research Institute, Abbasia, Cairo, Egypt) was used. The Vaccine was reconstituted in 2 ml sterile PBS (pH 7.4), titrated on the chorioallantoic membrane of 10-day-old specific pathogen free embryonated chicken eggs (Nile SPF, Koom Oshiem, Fayoum, Egypt). Half of the stock virus preparation was inactivated using β propiolactone as previously described [23].

### Animal inoculation

Ninety mice were divided into three groups (30 mice per group). Mice in group 1 and 2 were inoculated with 10^7 ^EID_50_/0.2 ml of inactivated and attenuated SPPV vaccine, respectively by i.p. route of inoculation. Mice in group 3 were kept as a placebo and inoculated with sterile PBS by the same route.

### Analyses of IL-10 and SOD from cultured peritoneal macrophages

The peritoneal macrophages were collected 12 h post i.p. inoculation of SPPV by peritoneal lavage. Cells were washed twice with sterile PBS, incubated in 24-well plate (Costar, Cramlington, U.K.) at a concentration of 1 × 10^6 ^cells/ml in RPMI 1640 (Gibco Laboratories, Grand Islands, NY) for 4 h at 37°C in a 5% CO_2 _tension. Non adherent cells were removed by three repeated  washings and peritoneal macrophages incubated with lipopolysaccharide 1 μg/ml (Sigma Chemical Co., USA) for 48 h at 37°C. At the end of incubation time, culture supernatants were collected, clarified by low speed centrifugation at 250 g for 10 min, and kept at -20°C until processing for IL-10, using mouse IL-10 immunoassay kit (BioSource International Inc. USA) according to manufacture instructions. SOD activity was also assessed in peritoneal macrophages' culture supernatants by means of the inhibition of pyrogallol autoxidation as described [22]. One unit of SOD activity is defined as the amount of enzyme required to inhibit autoxidation by 50% at 25 °C.

### Splenocytes preparation

Proliferation assay was conducted at 12 h, 3, 6, 9, 12 days post SPPV inoculation. Spleens were aseptically removed and placed in ice cold sterile PBS, (pH 7.4). Each spleen was squeezed with a 5 ml syringe plunger to extrude cells. Cell suspensions were centrifuged at 250 g for 10 min. Pelleted cells were resuspended in 5 ml of lysing buffer (Tris 0.17 M and ammonium chloride 0.16 M, pH 7.2) and incubated at room temperature for 5 min to lyse the red blood cells. Cell suspensions were washed twice with PBS and cell viability determined using trypan blue dye exclusion method.

### Splenocyte Proliferation Assay

Splenocytes were suspended in RPMI-1640 containing 10% FCS. One hundred micro-liters of suspended cells (1 × 10^5 ^cells per 100 ul) were added to each well of 96-well microtiter plate (Costar, Cramlington, U.K.). Splenocytes were stimulated with PHA-P (Sigma, Chemical Co.USA) at a final concentration of 10 ug/well. Cell cultures were incubated for 48 h at 37°C with 5% CO_2 _tension. Splenocyte proliferation was measured using 3-(4,5-dimethylthiazol-2-yl)-2,5-diphenyl tetrazolium bromide (MTT) dye uptake method as described [32]. The lymphocyte blastogenesis was expressed as stimulation index (SI): SI (%) = A2-A0/A1-A0. A2 is the absorption of cultures with PHA-P; A1 is the absorption of cultures without mitogen; A0 is absorption of the blank (culture medium only).

### Analyses of IL-12 and SOD from cultured splenocytes

The splenic macrophages were incubated in 24-well plate at a concentration of 1 × 10^6 ^cell/ml in RPMI 1640 for 4 h at 37°C in a 5% CO_2 _tension. Non adherent cells were removed by three repeated washings and splenic macrophages were incubated with lipopolysaccharide 1 μg/ml for 48 h at 37°C. At the end of incubation time, culture supernatants were collected, and kept at -20°C until processing for IL-12 using mouse IL-12 immunoassay kit (BioSource International Inc. USA) according to manufacture instructions. The assay recognizes both natural and free p40 subunit. SOD was also assessed as previously described.

### Effect of SPPV on the immune response to CRBC

Mice in all groups were inoculated i.p. with 1 × 10^7 ^CRBC at day 7. Serum was obtained seven days after CRBC immunization, tested for agglutinins against CRBC by the microhaemagglutination test according to [36].

### Statistical analysis

Analysis of variance (ANOVA) test was done for differences between treated groups according to [29].

## List of Abbreviations

Ab, antibody; Ag, antigen; Ags, antigens; attenu., attenuated; APC, antigen presenting cell; CRBC, chicken red blood cells; DC, dendritic cell; Th; T-helper; IL, interleukin; inac., inactivated; INF, interferon; NK, natural killer cell; PHA-P, phytohaemagglutinin-P; SOD, superoxide dismutase; SPPV, sheeppox virus;SI, stimulation index; TD, T-dependent; TNF, tumour necrosis factor.

## Competing interests

The author(s) declare that they have no competing interests.

## Authors' contributions

Abu-El-Saad participated in the design of the study, carried out the work with the mice, assisted in experimental work and drafting of the manuscript. Abdel-Moneim conceived the study, designed and carried out the experimental work and drafted the manuscript. All authors read and approved the final manuscript.
